# Multidisciplinary team discussion results in survival benefit for patients with stage III non-small-cell lung cancer

**DOI:** 10.1371/journal.pone.0236503

**Published:** 2020-10-08

**Authors:** Hsiu-Ying Hung, Yen-Han Tseng, Heng-Sheng Chao, Chao-Hua Chiu, Wen-Hu Hsu, Han-Shui Hsu, Yu-Chung Wu, Teh-Ying Chou, Chun-Ku Chen, Keng-Li Lan, Yi-Wei Chen, Yuan-Hung Wu, Yuh-Min Chen

**Affiliations:** 1 Nursing Department, Taipei Veterans General Hospital, Taipei, Taiwan; 2 Department of Chest Medicine, Taipei Veterans General Hospital, Taipei, Taiwan, Republic of China (R.O.C); 3 School of Medicine, National Yang-Ming University, Taipei, Taiwan, R.O.C; 4 Division of Thoracic Surgery, Department of Surgery, Taipei Veterans General Hospital, Taipei, Taiwan, R.O.C; 5 Division of Molecular Pathology, Department of Pathology and Laboratory Medicine, Taipei Veterans General Hospital, Taipei, Taiwan, R.O.C; 6 Department of Radiology, Taipei Veterans General Hospital, Taipei, Taiwan, R.O.C; 7 Department of Oncology, Taipei Veterans General Hospital, Taipei, Taiwan; 8 Taipei Cancer Center, Taipei Medical University, Taipei, Taiwan, R.O.C; MD Anderson Cancer Center, UNITED STATES

## Abstract

**Background:**

The treatment for stage III non-small cell lung cancer (NSCLC) often involves multi-modality treatment. This retrospective study aimed to evaluate whether multidisciplinary team (MDT) discussion results in better patient survival.

**Materials and methods:**

MDT discussion was optional before February 2016 and was actively encouraged by the MDT committee beginning February 2016. We reviewed the medical charts and computer records of patients with stage III NSCLC between January 2013 and December 2018.

**Results:**

A total of 515 patients were included. The median survival of all the patients was 33.9 months (M). The median survival of patients who were treated after MDT discussion was 41.2 M and that of patients treated without MDT discussion was 25.7 M (p = 0.018). The median survival of patients treated before February 2016 was 25.7 M and that of patients treated after February 2016 was 33.9 M (p = 0.003). The median survival of patients with stage IIIA tumors and those with stage IIIB tumors was 39.4 M and 25.7 M, respectively (p = 0.141). Multivariate analysis showed that MDT or not (p<0.001), T staging (p = 0.009), performance status (p<0.001), and surgery (p = 0.016) to be significant prognostic factors.

**Conclusion:**

The results of the study show that MDT discussion results in survival benefit in patients with stage III NSCLC. The MDT discussion, performance status, and if surgery was performed were independent prognostic factors for patients with stage III NSCLC.

## Introduction

Stage III non-small cell lung cancer (NSCLC) is a heterogeneous disease. It ranges from T1-T4, N0-N3, and resectable to unresectable. As a result treatment can vary widely from patient to patient. The treatment options include resection, neoadjuvant chemotherapy, adjuvant chemotherapy, concurrent chemo-radiotherapy (CCRT), and immunotherapy [1, 2]. Previous studies have proven CCRT to be superior to sequential chemoradiation therapy [3, 4]. Currently, definitive CCRT is considered standard of care for patients with stage IIIB NSCLC [5]. There is some controversy regarding the treatment paradigm for stage IIIA patients and thus the importance of multidisciplinary discussion. Treatment of lung cancer has progressed substantially in recent years, including targeted therapy and immunotherapy [6, 7]. The PACIFIC trial concluded CCRT followed by immunotherapy improved outcomes for patients with unresectable stage III NSCLC [8]. In addition, several trials that incorporate immunotherapy into neoadjuvant CCRT are ongoing at the time of this study [9]. ADJUVANT/CTONG1104 found gefitinib to be superior to cisplatin plus vinorelbine as adjuvant treatment for operable non-small cell lung cancer [10]. However, the best treatment strategy for stage III NSCLC has not been determined. Because of this, physicians and other specialists typically have discussions on a case-by-case basis to decide the best strategy for patients with stage III NSCLC. Decisions for the best strategy are based on findings from previous studies, as well as consideration for all available treatment options.

A multidisciplinary team (MDT) is a group of experts that aim to improve the treatment, quality of life, and outcomes for each patient. The concept began in 1970 when a group of specialists met to discuss their patient; this specific format came to be known as a tumor board [11]. MDT discussions offer many advantages, such as more precise diagnosis, shorter time from diagnosis to treatment, increased likelihood of administering all treatments, improved communication from all MDT members, and greater support when management must deviate from guidelines. On the other hand, there are also disadvantages, such as potential delay diagnosis of some patients, inadequate information to facilitate discussion, conflicts of opinions from MDT members, and time required for appropriate meeting preparation [12]. In addition, in studies MDT discussion has improved the quality of life to a greater extent than palliative care [13]. Some studies have suggested MDT discussion may be beneficial for patients with unresectable lung cancer [14, 15]. However, few studies to date have evaluated the benefit of MDT discussion for patients with stage III lung cancer.

Therefore, the aim of the study was to prove MDT discussion could prolong the average time of survival for patients with stage III NSCLC.

## Materials and methods

### Study design and patients

The thoracic oncology MDT of Taipei Veterans General Hospital began holding MDT meeting from December of 2006. The members of thoracic oncology MDT included chest physicians, surgeons, medical oncologists, radiation oncologists, radiologists, nuclear medicine physicians, pathologists, nurses, psychologists, and dietitians. All the specialists met once a week to discuss cases of lung cancer, especially in patients whose condition were complicated or in those with stage III tumor. Any case was allowed to be discussed at any point during the course of treatment; examples of case scenarios include when it was difficult to make a diagnosis before treatment, when multiple treatment modalities were indicated during treatment, and when it was too difficult to evaluate the response after treatment. At the hospital of focus, MDT discussion was initiated by doctors in charge of the patients on an option basis before February 2016 and is was actively encouraged by MDT committee for stage III NSCLC patients beginning February 2016.

We retrospectively reviewed chart and computer record of stage III NSCLC patients from January 2013 to December 2018. The clinicopathological data were recorded, including age, gender, smoking status, Eastern Cooperative Oncology Group performance status (ECOG PS), clinical staging (T status, and N status), and histology. We also recorded whether or not the patients underwent surgical intervention. All data were fully anonymized before we initially access them. The study was approved by the Institutional Ethical Review Board of Taipei Veterans General Hospital (VGHIRB No.: 2019-07-056BC) and informed consent was not required according to our institutional guidelines.

### Efficacy evaluation

Chest computed tomography scan was performed 1 month before treatment for staging and every 2 to 3 months after treatment in order to confirm the treatment response. The World Health Organization (WHO) TNM staging version 7 was used in this study [16]. Types of responses were assessed according to Response Evaluation Criteria in Solid Tumors (RECIST version 1.1) [17]. Overall survival was measured from the date of initiation of first treatment to the date of death due to any cause or the last follow-up. Overall survival was censored for the patients who were still alive at the time of the last follow-up visit.

### Statistical analysis

All categorical variables were analyzed with ϰ^2^ tests. Mann-Whitney U test were used for continuous variables when comparing 2 groups. Median overall survival (OS) were calculated using the Kaplan-Meier method and compared by log-rank test. Cox-regression analysis was used for multivariate OS analysis. All statistical analyses were performed using SPSS software (version 18.0; SPSS Inc., Chicago, IL, USA).

## Results

### Patients

**Fig 1:**
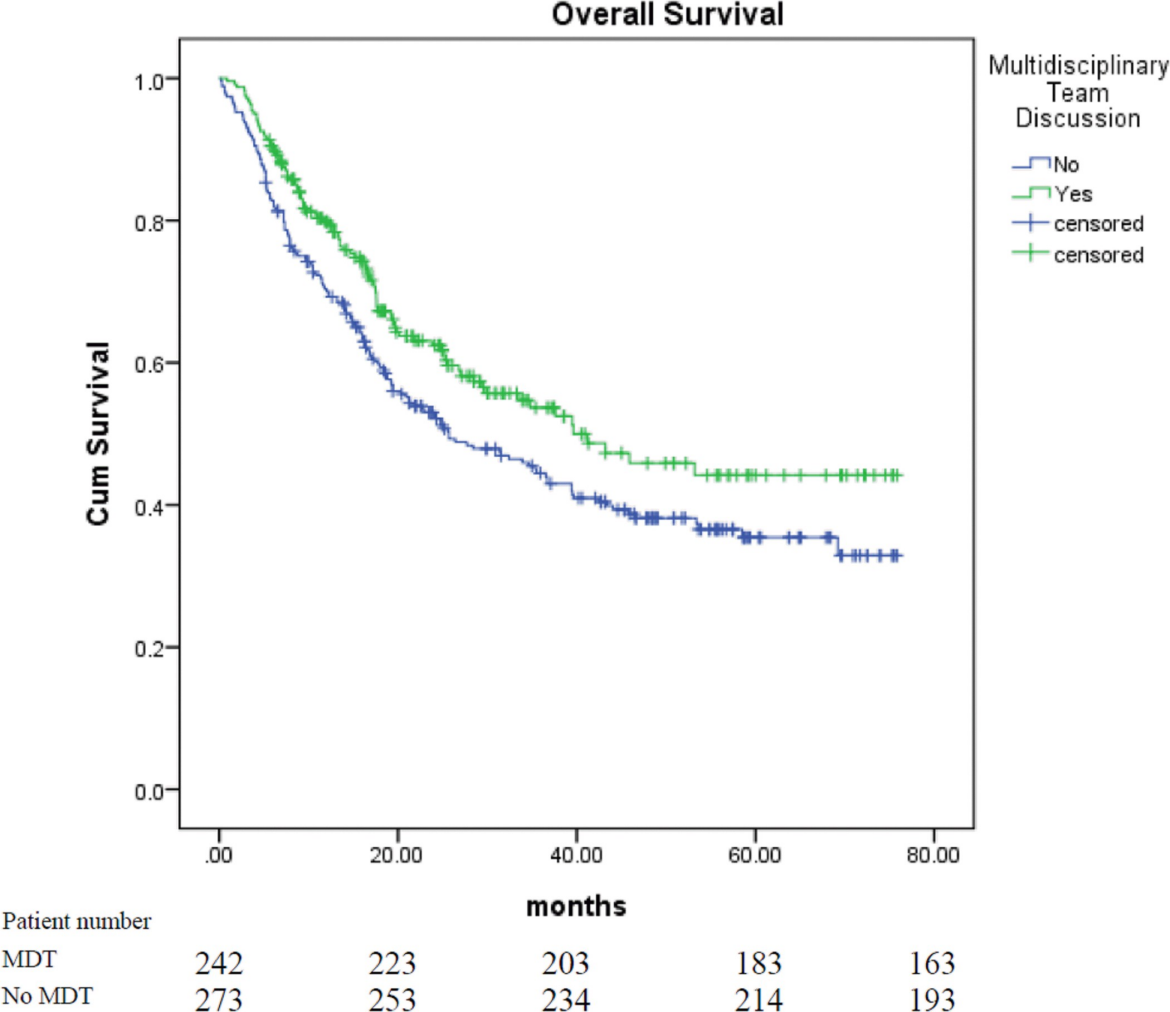
Survival curve of patients with stage III NSCLC according to whether MDT discussion was performed.

**Fig 2:**
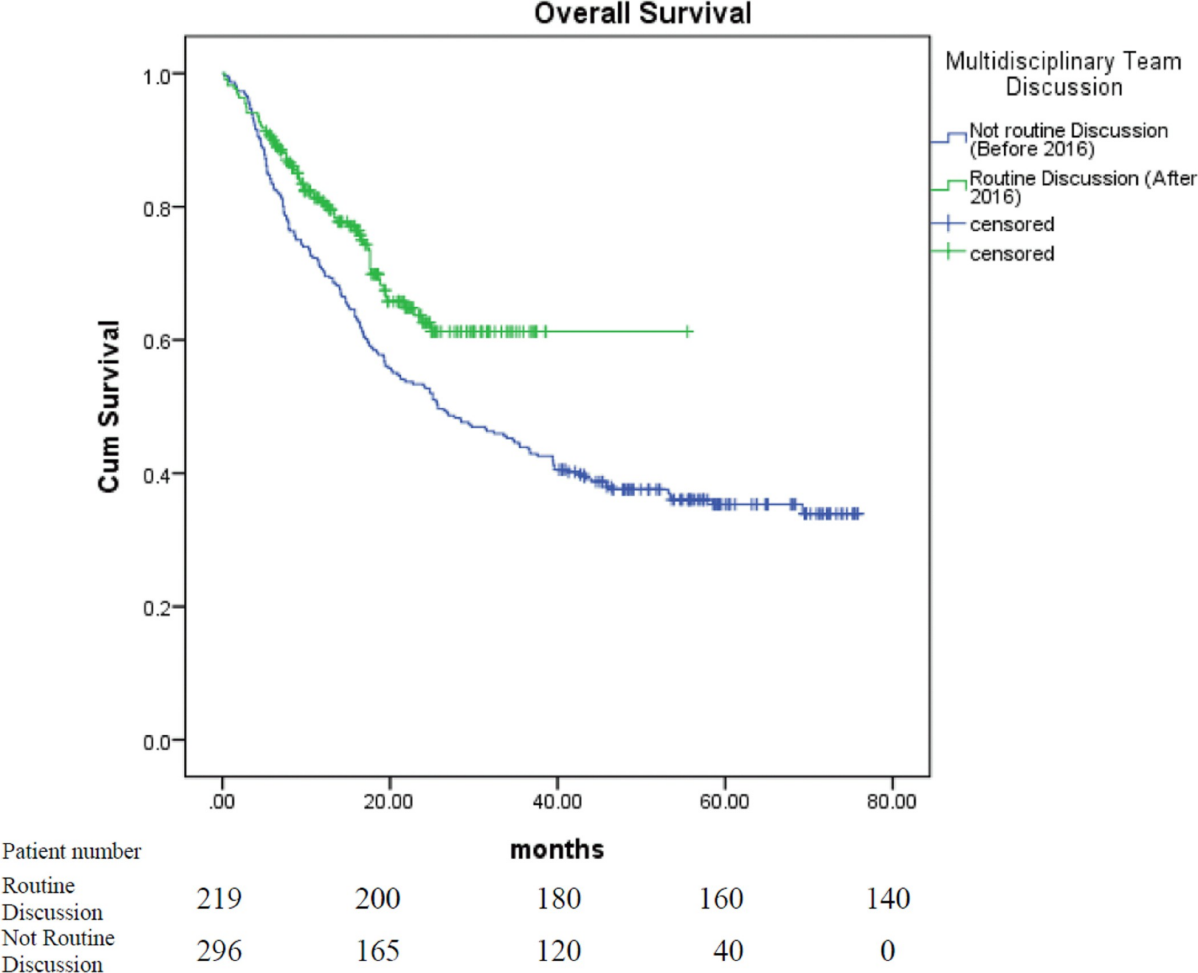
Survival curve of patients with stage III NSCLC treated before and after February 2016.

A total of 515 patients with stage III tumors were included in this study, of which 348 were men and 167 were women. The mean age was 68 years old, which ages ranging from 20 to 95 years old. The median follow up time was 34.30 months for patients received MDT and 23.83 months for patients who did not receive MDT. MDT discussion was performed for 39.4% of patients with stage III NSCLC between January 2013 and January 2016, and that increased to 69.3% of patients between February 2016 and December 2018 (p<0.001). The median length of survival of all the patients was 33.9 months (95% confidence interval [CI] 27.1–40.7). The median length of survival of patients who received treatment after MDT discussion (n = 235) was 41.2 months, as compared to 25.7 months for patients who received treatment without MDT discussion (n = 280; 95% CI 23–59.4, 17.1–34.3; p = 0.018, Fig 1). The median survival of all the patients treated before February 2016 was 25.7 months (n = 296, 95% CI 17.6–33.8), which increased to 33.9 months for patients treated after February 2016 (n = 219, 95% CI 27.1–40.7; p = 0.003, Fig 2). The median length of survival of male and female patients was 24.7 months (n = 348, 95% CI 19.7–29.7) and 53.4 months (n = 167, 95% CI—), respectively (p<0.001). The median survival of never smokers and smokers was not reached (n = 201, 95% CI—) and 21.2 months (n = 314, 95% CI 17.2–25.2), respectively (p<0.001). The median length of survival according to the PS was not reached (95% CI—) for patients with a PS score of 0 (n = 206), 24.9 months (95% CI 18.4–31.4) for patients with a PS score of 1 (n = 244), 8.6 months (95% CI 1.7–15.5) for those with a PS score of 2 (n = 42), 3.7 months (95% CI 2.1–5.3) for those with a PS score of 3 (n = 17), and 2.9 months (95% CI 0–6.3) for those with a PS score of 4 (n = 6; p<0.001). The median length of survival for patients with squamous cell carcinoma (n = 184), adenocarcinoma (n = 281), and other subtypes (n = 50) was 17.8 months (95% CI 15.6–20), 58.5 months (95% CI 38.6–78.4), and 21.3 months (95% CI 13.3–29.3), respectively (p<0.001). The median length of survival of patients with stage IIIA tumors (n = 276) and those with stage IIIB tumors (n = 239) was 39.4 months (95% CI 28.9–49.9) and 25.7 months (95% CI 15.9–35.5), respectively (p = 0.141). The median survival of patients who underwent surgery (n = 148) and those who did not was not reached (95% CI—) and 24 months (n = 367, 95% CI 19.7–28.3), respectively (p<0.001).

### Survival according to different TNM stages

Filtering data according to T status, the median survival for patients was not reached (95% CI—) in patients with T1 disease (n = 57), 39.6 months (95% CI—) in those with T2 disease (n = 146), 21.2 months (95% CI 16.8–25.6) in those with T3 disease (n = 159), and 26.4 months (95% CI 11.4–41.4) in patients with T4 disease (n = 153; p = 0.001). Filtering data according to N status, the median length of survival for patients with N0 disease (n = 32) was not reached (95% CI—), in patients with N1 disease (n = 67) was 26.8 months (95% CI 19–34.6), in patients with N2 disease (n = 246) was 37.6 months (95% CI 28.8–46.4), and in patients with N3 disease (n = 170) was 25.7 months (95% CI 13.2–38.2).

### Cox regression model analysis for all the factors

Multivariate analysis included whether or not MDT discussion was performed, sex, staging, T status, and N status, smoking, PS, histology, and whether surgery was performed. The results showed that MDT or not (p<0.001), T staging (p = 0.009), ECOG PS (p<0.001), and surgery (p = 0.016) were significant prognostic factors (Table 1). According to the result of multivariate analysis, MDT discussion prolonged length of survival of patients with stage III lung cancer.

**Table 1:** General data for patients from 2013 to 2018.

		*Patient number*	*Median survival (months)*	*95% CI*	*p value*	*Cox regression*
Before Feb 2016 vs later	before	296	25.7	17.6~33.8	0.003	N/A
	after	219	33.9	27.1~40.7		
MTD vs no MTD	yes	242	39.6	23~56.2	0.018	0.184
	no	273	25.7	27.1~40.7		
Gender	male	348	24.7	19.7~29.7	0.000	0.205
	female	167	53.4	NR		
Staging	IIIA	276	39.4	28.9~49.9	0.141	0.795
	IIIB	239	25.7	15.9~35.5		
	III	515	33.9	27.1~40.7		
T	1	57	NR	─,─	0.001	0.008
	2	146	39.6	─,─		
	3	159	21.2	16.8~25.6		
	4	153	26.4	11.4~41.4		
N	0	32	NR	─,─	0.928	0.131
	1	67	26.8	19~34.6		
	2	246	37.6	28.8~46.4		
	3	170	25.7	13.2~38.2		
smoking	no	201	NR	─,─	0.000	0.051
	yes	314	21.2	17.2~25.2		
ECOG PS	0	206	NR	─,─	0.000	0.000
	1	244	24.9	18.4~31.4		
	2	42	8.6	1.7~15.5		
	3	17	3.7	2.1~5.3		
	4	6	2.9	0~6.3		
surgery	yes	148	NR	─,─	0.000	0.023
	no	367	24	19.7~28.3		

MDT: multi-disciplinary team; PS: performance status; NR: not reached.

## Discussion and conclusion

The current study is to demonstrate MDT discussion prolongs the survival time of patients with stage III NSCLC. In fact, previous studies have shown poor outcomes for patients with stage III NSCLC. The 5 year survival rate is approximately 15–30% [2, 18]. Although some clinical trials have used consolidation chemotherapy after standard CCRT, they have still failed to prolong the PFS [19, 20]. In previous studies, the median length of survival was 18–23 months [21, 22]. In our study, the median length of survival was 25.7 months in patients who received treatment without MDT discussion, and this result was similar to the length of survival obtained in previous studies. However, the median length of survival time in our study was 41.2 months when patients received treatment after MDT discussion. This result suggests that MDT prolongs the length of survival for patients with stage III NSCLC. In addition, the median length of survival is approximately 22–24 months for patients with stage IIIA disease and 12–15 months for patients with stage IIIB disease [23–26]. The current study found the median survival was 39.4 months for patients with stage IIIA disease and 25.7 months for patients with stage IIIB disease. Thus, the median length of survival was longer for patients included in the current study than for patients in previous studies. The recently PACIFIC trial is among the most commonly cited studies that enrolled patients with stage III, unresectable NSCLC. The median length of survival of patients in the placebo group in PACIFIC trial was 16.2 months [8]. Meanwhile, the median length of survival of patients who did not undergo surgery was 24 months. Thus, MDT discussion seems to play a key role in prolonging the survival of patients with stage III NSCLC.

MDT discussion began in our hospital in 2007. MDT discussion was performed for only 39.4% of patients with stage III NSCLC patients between January 2013 and January 2016. In 2015, Kehl KL et al. concluded that MDT should focus on complex cases because combined modality treatment are more likely to provide benefit for lung cancer patients [27]. In the current study, MDT discussion was performed for patients with stage III NSCLC; 69.3% of patients with stage III lung cancer were treated after MDT discussion between February 2016 and December 2018. The median length of survival of all the patients treated before February 2016 was 25.7 months (n = 296, 95% C.I. 17.6–33.8), and it increased to 33.9 months for patients treated after February 2016 (n = 219, 95% C.I. 27.1–40.7) (p = 0.003).

The findings of the current study showed that the survival of female patients was better than that of male patients. The influence of sex on survival of patients with NSCLC is still unclear and results are inconclusive. While some studies have concluded that sex may not affect the survival of patients with resectable NSCLC [28, 29], others have shown male gender to be an unfavorable prognostic factor for non-small cell lung cancers [30, 31]. But, the reason for the difference in results is not clear. Pinto et al. indicated female patients may benefit more from targeted therapy. In addition, more male patients are smokers than female patients [32]. In the current study we did not check EGFR status for every patient. Therefore more data are warranted in order to make a conclusion.

In the current study, never smokers had a longer survival than smokers. Smoking increases not only the risk of lung cancer but also the rate of recurrence [33]. Moreover, smoking appears to decrease the response to cancer treatment [34–36]. Continuing smoking after diagnosis of lung cancer is known to increase the risk all-cause mortality for patients [33]. Therefore, smoking cessation is necessary for effective lung cancer treatment [37–39]. We recommend MDT discussion should also include a program for smoking cessation as a part of lung cancer care.

In previous studies PS has been associated with length of survival for patients with NSCLC [40, 41]. It is known that PS would influence a physician’s decision regarding the appropriateness of chemotherapy [42]. Data in the current study revealed patients with better PS had longer length survival. In addition, MDT discussion did not result in a more aggressive treatment plan. In our study, 23 patients had a PS score of 3–4, and most of them received supportive care or palliative radiotherapy regardless of whether or not there were MDT discussions.

TNM staging is the most important predictor for length of survival for patients with lung cancer. IASLC published the AJCC 7 staging manual and its results showed the median length of survival was not reached, 113 months, 81 months, 56 months, and 29 months when the tumor size was <2 cm (T1a), 2–3 cm (T1b), 3–5 cm (T2a), 5–7 cm (T2b), and >7 cm (T3), respectively, among patients with stage I-IV NSCLC [43]. In the current study, the median length of survival was not reached for patients with T1 disease, 39.6 months for those with T2 disease, 21.2 months for those with T3 disease, and 26.4 months for those with T4 disease. Thus, the survival of patients with the same T stage was still worse for patients with stage III NSCLC.

There are some limitations about this study that should be mentioned. First, this was a retrospective study, so there was undoubtedly some selection bias. A large, prospective, randomized trial is necessary to achieve definite answers to the questions raised. Second, Due to differences in patient characteristics, physicians might choose different chemotherapy regimens, which could confound the outcome of the study. Finally, treatment options increase and guidelines change as time goes by. These factors could influence outcomes of patients with lung cancer.

In conclusion, MDT discussion is essential given current treatment options and efforts to maximize effectiveness. Although treatment for patients with stage III NSCLC is complicated, MDT discussion prolongs their survival time and should be actively performed.

## Supporting information

S1 Raw Data (XLSX)
